# Optimal Geometries for AOA Localization in the Bayesian Sense

**DOI:** 10.3390/s22249802

**Published:** 2022-12-14

**Authors:** Kutluyil Dogancay

**Affiliations:** UniSA STEM, University of South Australia, Mawson Lakes, SA 5095, Australia; kutluyil.dogancay@unisa.edu.au

**Keywords:** optimal target–sensor geometries, bearings-only localization, Fisher information matrix, Bayesian estimation

## Abstract

This paper considers the optimal sensor placement problem for angle-of-arrival (AOA) target localization in the 2D plane with a Gaussian prior. Optimal sensor locations are analytically determined for a single AOA sensor using the D- and A-optimality criteria and an approximation of the Bayesian Fisher information matrix (BFIM). Optimal sensor placement is shown to align with the minor axis of the prior covariance error ellipse for both optimality criteria. The approximate BFIM is argued to be valid for a sufficiently small prior covariance compared with the target range. Optimal sensor placement results obtained for Bayesian target localization are extended to manoeuvring target tracking. For sensor trajectory optimization subject to turn-rate constraints, numerical search methods based on the D- and A-optimality criteria as well as a new closed-form projection algorithm that aims to achieve alignment with the minor axis of the prior error ellipse are proposed. It is observed that the two optimality criteria generate significantly different optimal sensor trajectories despite having the same optimal sensor placement for the localization of a stationary target. Analysis results and the performance of the sensor trajectory optimization methods are demonstrated with simulation examples. It is observed that the new closed-form projection algorithm achieves superior tracking performance compared with the two numerical search methods.

## 1. Introduction

In target tracking and localization problems, target–sensor geometries are known to play a significant role in determining the localization and tracking performance. In this paper, we focus on optimal target–sensor geometries for angle-of-arrival (AOA) localization in the 2D plane using a single moving sensor. First, the localization problem is cast as a Bayesian estimation problem, which assumes the availability of prior information in the form of a Gaussian prior for the unknown target location. For this problem, optimal sensor placement results are developed using approximate estimation bounds. Next, the Bayesian estimation problem is extended to target tracking using the Kalman filter, and optimal sensor trajectories are developed to track a manoeuvring target.

Optimal sensor placement has been researched for several decades. Early works included [1,2,3], where the performance of the extended Kalman filter (EKF) [4] and several deterministic (non-Bayesian) estimators was reported for different bearings-only sensor manoeuvres, mostly in sonar applications. In [5], optimal bearings-only sensor manoeuvres for tracking a constant-velocity target were derived using optimal control theory. The sensor trajectory optimization problem was formulated as a partially observable Markov decision problem (POMDP) for a manoeuvring target using the trace of FIM, which is similar to the A-optimality criterion (minimizing trace of inverse FIM), as the reward function in [6]. In [7], the D-optimality criterion, whereby the sensor location is determined to maximize the determinant of the Fisher information matrix (FIM), was adopted to determine optimal sensor trajectories for the localization of a stationary target. Optimal sensor manoeuvres necessary to make a constant-velocity target observable were discussed in [8]. The sensor trajectory optimization problem in the Bayesian sense was considered in [9], where the posterior Cramer–Rao lower bound (PCRLB) [10] was employed to minimize the largest root-mean-square-error (RMSE)-bound approximated by the reciprocal of measurement data contributions to target location information.

A comprehensive analysis of the 2D optimal AOA sensor placement problem for a stationary target was presented in [11,12]. Optimal 3D AOA target–sensor geometries for a stationary target were analysed in [13]. A gradient-descent algorithm for sensor path optimization to minimize the mean-square error of predicted EKF target location estimates was proposed in [14]. A UAV path optimization algorithm that solves a nonlinear programming problem based on the D-optimality criterion was developed in [15] to geolocate a stationary target using a heterogeneous mix of passive payload sensors. In [16], a unified framework was proposed for AOA, range-only, and received signal strength localization when the target was stationary. Optimal target–sensor geometries for maximum a posteriori (MAP) target localization with a Gaussian prior were investigated in [17]. In [18], the optimality criteria for target–sensor geometries in a Kalman filtering setting were analysed for several sensor types using an approximation of the Bayesian FIM. A unified 2D target–sensor geometry optimization framework was proposed in [19] for stationary target localization with a Gaussian prior, reducing the optimization problem to minimization of the modulus of a vector sum, akin to [11]. In [20], optimal sensor placement in 3D space was studied for AOA target localization with a Gaussian prior, employing rotational invariance arguments.

This paper develops optimal sensor placement results for a single AOA sensor at a fixed distance from the mean of the Gaussian prior. To do this, the Bayesian FIM is approximated by replacing the expectation of the contribution form measurement data with its instantaneous value calculated at the mean of the Gaussian prior. It is argued that this approximation is valid when the covariance of the Gaussian prior is relatively small compared with the target range. The optimal sensor placements for the D- and A-optimality criteria are shown to be identical and align with the minor axis of the error ellipse of the prior covariance. In the context of bearings-only manoeuvring target tracking, numerical search methods based on the D- and A-optimality criteria and a new closed-form projection algorithm that attempts to achieve alignment with the minor axis of the prior covariance error ellipse are proposed for sensor trajectory optimization subject to turn-rate constraints. It is observed that the D- and A-optimality criteria yield markedly different optimal sensor trajectories even though they produce identical optimal sensor placement for the localization of a stationary target. The projection algorithm is shown to outperform the other two methods in simulation studies.

This paper is organized as follows. Section 2 investigates the optimal sensor placement problem for a stationary target with a Gaussian prior using the D- and A-optimality criteria. Section 3 extends the results of Section 2 to Kalman filter tracking of a manoeuvring target, proposing two sensor trajectory optimization methods and a new closed-from projection algorithm. Section 4 presents simulation examples to verify the optimal sensor placement results derived in Section 2 and to compare and demonstrate the effectiveness of the sensor trajectory optimization algorithms proposed in Section 3. Concluding remarks are made in Section 5.

## 2. Optimal Target-Sensor Geometry with Gaussian Prior

**Figure 1:**
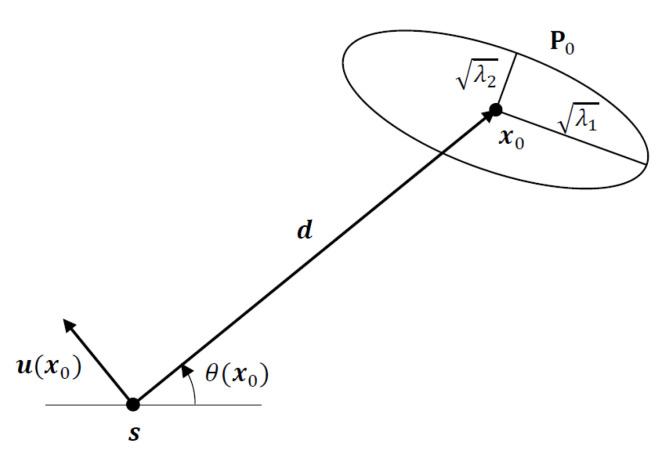
The 2D AOA geometry and 1-σ error ellipse for Gaussian prior with mean $x_{0}$ and covariance $P_{0}$.

In tracking problems with a state space that can be modelled or approximated as a Gauss–Markov process, the Kalman filter has been extensively used to compute the Gaussian prior for state estimates from the noisy sensor measurements available in each recursion in the form of the predicted state estimate and predicted state covariance. Starting with the Gaussian prior $N(x_{0},P_{0})$, where $x_{0}$ is the mean and $P_{0}$ is the covariance of the prior, the objective of optimal sensor placement is to determine a sensor location s at a fixed distance of d=∥d∥, where $d=x_{0}-s$, from the mean of the Gaussian prior (or predicted target location estimate) $x_{0}$ so that sensor measurements collected at the new sensor location will optimize a well-defined objective function that is related to the Bayesian localization performance. The new measurements together with the Gaussian prior are then used to compute the filtered state estimate and covariance, which are optimized in terms of target–sensor geometry. We consider two optimality criteria for sensor placement: namely, the D-optimality and A-optimality criteria [21,22,23], which are commonly used in practice. Referring to Figure 1, the task of geometry optimization is reduced to finding a range vector d or bearing angle $\theta (x_{0})$ pivoted at the mean of the Gaussian prior with d=∥d∥ fixed, which gives the location of the sensor to satisfy the chosen optimization criterion.

The bearing measurements collected by the sensor located at s are given by

(1) θ=θ(x)+w

where $x∼N(x_{0},P_{0})$ is the Bayesian prior, $w∼N(0,\sigma^{2})$ is the bearing angle noise, and

(2) $$\theta \left(x\right)=\tan^{-1}\left(x_{2}-s_{2},x_{1}-s_{1}\right),\;-\pi \leq h\left(x\right)\leq \pi$$

is the true bearing angle with $x={\left[x_{1},x_{2}\right]}^{T}$ and $s={\left[s_{1},s_{2}\right]}^{T}$. In (2), $\tan^{-1}\left(\cdot \right)$ is the four-quadrant arc-tangent. The objective of Bayesian estimation is to determine an estimate for the unknown random target location x from the bearing measurement θ and the knowledge of the Gaussian prior with mean $x_{0}$ and covariance $P_{0}$.

The optimality criteria considered in this paper employ estimation bounds obtained from the FIM or CRLB. In Bayesian estimation problems that involve random unknown parameters, these bounds are replaced by the Bayesian FIM (BFIM) or Bayesian CRLB (BCRLB). The BFIM for the single-sensor AOA localization problem is defined as [24]

(3) $$\Phi =K_{0}+E_{x}\left\{\frac{1}{\sigma^{2}d^{2}\left(x\right)}u\left(x\right)u^{T}\left(x\right)\right\}$$

where $E_{x}\left\{\cdot \right\}$ denotes the expectation over x,

(4) $$u\left(x\right)=\left[\begin{array}{c} -\sin\theta (x)\\ \cos\theta (x) \end{array}\right]$$

is the unit vector orthogonal to the range vector, and d(x)=∥x−s∥ is the target range from the sensor positioned at s. The matrix $K_{0}=P_{0}^{-1}$ represents the contribution of the a priori information, and

(5) $$E_{x}\left\{\frac{1}{\sigma^{2}d^{2}\left(x\right)}u\left(x\right)u^{T}\left(x\right)\right\}$$

is the contribution of data. The inverse of the BFIM gives the BCRLB.

Equation (5) can be rewritten as

(6) $$\begin{array}{cc} E_{x}\left\{\frac{1}{\sigma^{2}d^{2}\left(x\right)}u\left(x\right)u^{T}\left(x\right)\right\} & =\frac{1}{\sigma^{2}}E_{x}\left\{\frac{1}{d^{2}\left(x\right)}u\left(x\right)u^{T}\left(x\right)\right\} \end{array}$$

(7) $$\begin{array}{cc} \; & =\frac{1}{\sigma^{2}}\left[\begin{array}{cc} E\left\{\frac{\sin^{2}\theta \left(x\right)}{{∥x-s∥}^{2}}\right\} & -E\left\{\frac{\sin\theta (x)\cos\theta (x)}{{∥x-s∥}^{2}}\right\}\\ -E\left\{\frac{\sin\theta (x)\cos\theta (x)}{{∥x-s∥}^{2}}\right\} & E\left\{\frac{\cos^{2}\theta \left(x\right)}{{∥x-s∥}^{2}}\right\} \end{array}\right] \end{array}$$

(8) $$\begin{array}{cc} \; & \approx \frac{1}{\sigma^{2}d^{2}}u\left(x_{0}\right)u^{T}\left(x_{0}\right),\;u\left(x_{0}\right)=\left[\begin{array}{c} -\sin\theta (x_{0})\\ \cos\theta (x_{0}) \end{array}\right] \end{array}$$

which gives

(9) $$\Phi \approx K_{0}+\frac{1}{\sigma^{2}d^{2}}u\left(x_{0}\right)u^{T}\left(x_{0}\right).$$

**Figure 2:**
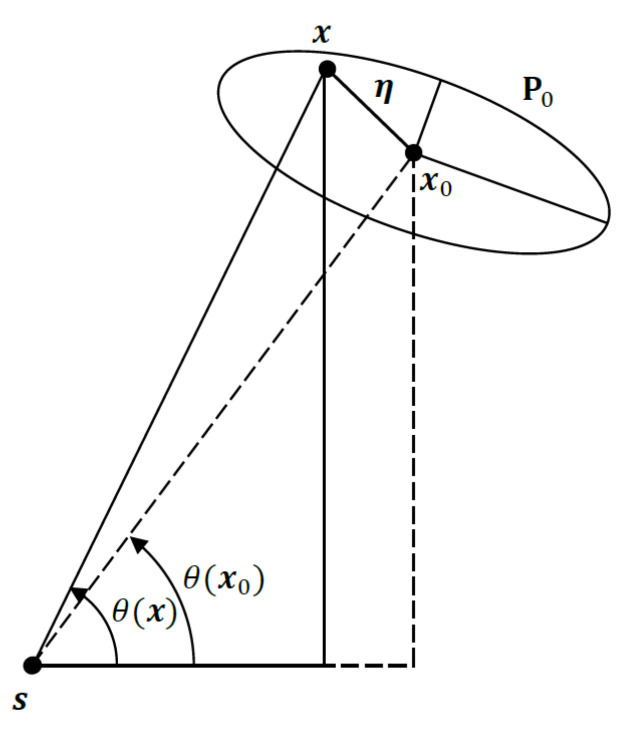
Geometric interpretation of instantaneous angle measurements.

The approximation in (9) is valid for a sufficiently “small” prior covariance compared with the target range. Here, we measure the size of prior covariance by its trace (see (16)). Referring to Figure 2, we have

(10) $$\begin{array}{cc} \sin\theta (x) & =\sin\theta (x_{0}+\eta ) \end{array}$$

(11) $$\begin{array}{cc} \; & =\frac{x_{0,2}-s_{2}+\eta_{2}}{\sqrt{{\left(x_{0,1}-s_{1}+\eta_{1}\right)}^{2}+{\left(x_{0,2}-s_{2}+\eta_{2}\right)}^{2}}} \end{array}$$

where $\eta ={\left[\eta_{1},\eta_{2}\right]}^{T}∼N\left(0,P_{0}\right)$, and $x_{0}={\left[x_{0,1},x_{0,2}\right]}^{T}$. Taking the expectation of the squared sine function yields

(12) $$\begin{array}{cc} E\{\sin^{2}\theta \left(x\right)\} & \approx \frac{E\{{\left(x_{0,2}-s_{2}+\eta_{2}\right)}^{2}\}}{E\{{\left(x_{0,1}-s_{1}+\eta_{1}\right)}^{2}+{\left(x_{0,2}-s_{2}+\eta_{2}\right)}^{2}\}} \end{array}$$

(13) $$\begin{array}{cc} \; & \approx \frac{{\left(x_{0,2}-s_{2}\right)}^{2}+E\left\{\eta_{2}^{2}\right\}}{d^{2}+E\left\{\eta_{1}^{2}\right\}+E\left\{\eta_{2}^{2}\right\}} \end{array}$$

(14) $$\begin{array}{cc} \; & \approx {\left(\frac{x_{0,2}-s_{2}}{d}\right)}^{2} \end{array}$$

(15) $$\begin{array}{cc} \; & \approx \sin^{2}\theta \left(x_{0}\right) \end{array}$$

if

(16) $$d^{2}\gg E\left\{\eta_{1}^{2}\right\}+E\left\{\eta_{2}^{2}\right\}=\mathrm{tr}P_{0}.$$

Here, tr denotes trace. Applying the same line of reasoning to E{sinθ(x)cosθ(x)} and $E\{\cos^{2}\theta \left(x\right)\}$, we conclude that (16) is the condition that must be met to justify (9).

### 2.1. D-Optimality Criterion

The *D*-optimality criterion aims to maximize the determinant of the Fisher information matrix (FIM). For the Bayesian estimation problem considered here, the FIM is replaced by the Bayesian FIM (BFIM). Considering the optimization problem described in Figure 1, the optimal placement for the sensor is obtained from the solution of

(17) $$\max_{s}\;\left|\Phi \right|$$

where |·| denotes the determinant. The optimization problem in (17) determines the sensor location s that maximizes the determinant of BFIM for a given Gaussian prior.

Noting that in (9) $K_{0}$ is a square matrix and $u(x_{0})$ is a column vector, the determinant of the BFIM can be rewritten as a sum of two terms [25]

(18) $$\begin{array}{cc} \left|\Phi \right| & \approx \left|K_{0}+\frac{1}{\sigma^{2}d^{2}}u\left(x_{0}\right)u^{T}\left(x_{0}\right)\right| \end{array}$$

(19) $$\begin{array}{cc} \; & \approx |K_{0}|+\frac{1}{\sigma^{2}d^{2}}u^{T}\left(x_{0}\right)K_{0}^{*}u\left(x_{0}\right) \end{array}$$

where $K_{0}^{*}$ is the adjoint of $K_{0}$, defined by

(20) $$K_{0}^{*}=|K_{0}|K_{0}^{-1}=\left|K_{0}\right|P_{0}.$$

Thus, for a given Gaussian prior $P_{0}$ and fixed *d*, the optimization problem in (17) reduces to

(21) $$\max_{\theta (x_{0})}\;u^{T}\left(x_{0}\right)P_{0}u\left(x_{0}\right).$$

**Figure 3:**
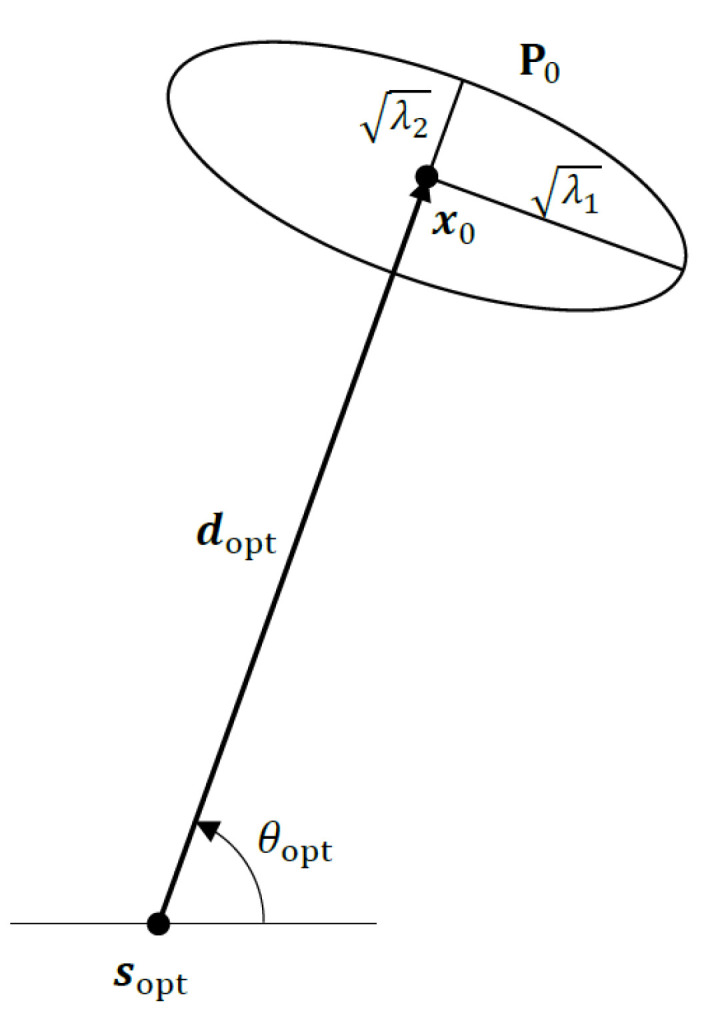
Optimal sensor placement in 2D using the D-optimality criterion with Gaussian prior $N(x_{0},P_{0})$.

Since $u(x_{0})$ is a unit vector, (21) is a problem of quadratic form maximization over the unit circle. Using the eigenvalues of $P_{0}$, denoted by $\lambda_{1},\lambda_{2}$ with $\lambda_{1}\geq \lambda_{2}$, the solution of (21) is given by [26]:

(22) $$\begin{array}{c} \lambda_{1}=\max_{\theta (x_{0})}\;u^{T}\left(x_{0}\right)P_{0}u\left(x_{0}\right) \end{array}$$

(23) $$\begin{array}{c} \nu_{1}=\underset{u(x_{0})}{arg max}\;u^{T}\left(x_{0}\right)P_{0}u\left(x_{0}\right) \end{array}$$

where $\nu_{1}$ is an orthonormal eigenvector of $P_{0}$ corresponding to $\lambda_{1}$. The optimal bearing angle for the sensor, $\theta_{\mathrm{opt}}$, is easily obtained from the optimal unit vector $\nu_{1}$ by noting that it is orthogonal to the range vector (see Figure 1). In other words, the optimal range vector $d_{\mathrm{opt}}$ must be aligned with the minor axis of the error ellipsoid of the Gaussian prior, as shown in Figure 3.

Some remarks are in order here:

- If the Gaussian prior has a circular error ellipse with $P_{0}$ given by a scaled identity matrix, (21) becomes
  (24) $$\max_{\theta (x_{0})}\;u^{T}\left(x_{0}\right)u\left(x_{0}\right)\;\mathrm{or}\;\max_{\theta (x_{0})}\;1$$
  which means that optimality is achieved by any bearing angle $\theta (x_{0})$.
- In all other cases, there are two optimal bearing angles aligned with the minor axis of the error ellipse, producing two possible optimal sensor locations with range vectors $\pm d_{\mathrm{opt}}$ symmetric about $x_{0}$.

### 2.2. A-Optimality Criterion

The objective of the *A*-optimality criterion is to minimize the trace of the BCRLB or the inverse BFIM. In this case, the optimal sensor placement is obtained from

(25) $$\min_{s}\;\mathrm{tr}\left(\Phi^{-1}\right).$$

Applying the matrix inversion lemma [27] to the approximate BFIM in (9), we get

(26) $$\Phi^{-1}\approx P_{0}\left(I-\frac{u\left(x_{0}\right)u^{T}\left(x_{0}\right)P_{0}}{\sigma^{2}d^{2}+u^{T}\left(x_{0}\right)P_{0}u\left(x_{0}\right)}\right)$$

It is clear that to solve (25), we need to maximize the trace of the second term on the right-hand side of (26); i.e.,

(27) $$\begin{array}{c} \max_{s}\mathrm{tr}\left(\frac{P_{0}u\left(x_{0}\right)u^{T}\left(x_{0}\right)P_{0}}{\sigma^{2}d^{2}+u^{T}\left(x_{0}\right)P_{0}u\left(x_{0}\right)}\right) \end{array}$$

or

(28) $$\begin{array}{c} \max_{u(x_{0})}\frac{u^{T}\left(x_{0}\right)P_{0}^{2}u\left(x_{0}\right)}{\sigma^{2}d^{2}+u^{T}\left(x_{0}\right)P_{0}u\left(x_{0}\right)}. \end{array}$$

To solve (28) for $u(x_{0})$, let $R=\frac{u^{T}\left(x_{0}\right)P_{0}^{2}u\left(x_{0}\right)}{\sigma^{2}d^{2}+u^{T}\left(x_{0}\right)P_{0}u\left(x_{0}\right)}$ and set its gradient equal to zero,

(29) $$\frac{\partial }{\partial u(x_{0})}R=0$$

which results in

(30) $$\begin{array}{c} \frac{2P_{0}^{2}u\left(x_{0}\right)\left(\sigma^{2}d^{2}+u^{T}\left(x_{0}\right)P_{0}u\left(x_{0}\right)\right)-2\left(u^{T}\left(x_{0}\right)P_{0}^{2}u\left(x_{0}\right)\right)P_{0}u\left(x_{0}\right)}{{\left(\sigma^{2}d^{2}+u^{T}\left(x_{0}\right)P_{0}u\left(x_{0}\right)\right)}^{2}}=0 \end{array}$$

(31) $$\begin{array}{c} P_{0}^{2}u\left(x_{0}\right)\left(\sigma^{2}d^{2}+u^{T}\left(x_{0}\right)P_{0}u\left(x_{0}\right)\right)=\left(u^{T}\left(x_{0}\right)P_{0}^{2}u\left(x_{0}\right)\right)P_{0}u\left(x_{0}\right) \end{array}$$

(32) $$\begin{array}{c} P_{0}^{2}u\left(x_{0}\right)=RP_{0}u\left(x_{0}\right). \end{array}$$

Left-multiplying both sides of the above equation with $P_{0}^{-1}$ finally gives

(33) $$P_{0}u\left(x_{0}\right)=Ru\left(x_{0}\right)$$

where the nonzero scalar *R* is an eigenvalue of $P_{0}$ and the unit vector $u(x_{0})$ is the corresponding eigenvector. We therefore conclude that *R* is maximized when $u\left(x_{0}\right)=\nu_{1}$, which is the eigenvector of $P_{0}$ associated with its largest eigenvalue $\lambda_{1}$. Note that this optimality result is identical to that for the A-optimality criterion derived in Section 2.1.

## 3. Application to Tracking

In this section, sensor waypoint optimization algorithms are devised to embed the optimal sensor placement results derived in Section 2 into the Kalman filter. As a specific application, bearings-only manoeuvring target tracking is considered. When the target is moving, it is often the case that the target dynamics and constraints on the motion of a single sensor, such as turn-rates and distances between successive waypoints, do not allow strictly optimal sensor placement geometries to be achieved from one Kalman filter recursion to the next. We develop sensor trajectory optimization methods that respect dynamic sensor constraints.

**Figure 4:**
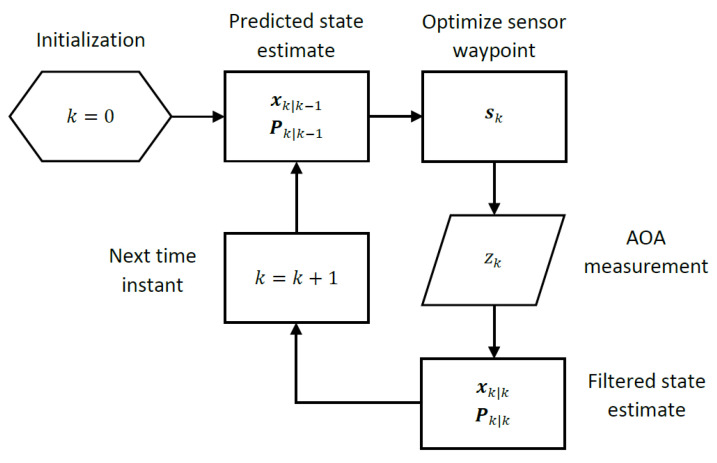
Optimal sensor waypoint computation in a Kalman filter recursion.

The principle we follow is based on the treatment of each Kalman filter recursion as solving a Bayesian target localization problem with a Gaussian prior available from the previous recursion and measurements taken at a new optimized sensor location to compute filtered state estimates and an updated prior for the next Kalman filter recursion. Figure 4 captures the computational steps of a Kalman filter recursion with sensor waypoint optimization embedded into it. The details of how optimal sensor waypoints are computed from Kalman filter parameters are discussed later in the section.

The single moving sensor collects AOA measurements from a manoeuvring target at time instants k=0,1,2,…. The sensor location at time *k* is denoted by $s_{k}$. The process equation for the target is

(34) $$x_{k+1}=Fx_{k}+n_{k},\;k=0,1,\ldots$$

where $x_{k}={\left[x_{k},{\dot{x}}_{k},y_{k},{\dot{y}}_{k}\right]}^{T}$ is the target state vector with ${\left[x_{k},y_{k}\right]}^{T}$ and ${\left[{\dot{x}}_{k},{\dot{y}}_{k}\right]}^{T}$ denoting the target location and velocity, respectively, at time *k*. In (34) the dynamical constraint (the state transition matrix) is given by

(35) $$F=\left[\begin{array}{cc} A & 0\\ 0 & A \end{array}\right],\;A=\left[\begin{array}{cc} 1 & T\\ 0 & 1 \end{array}\right]$$

where *T* denotes the time interval between discrete-time instants *k*. The process noise $n_{k}$ accounts for unknown target manoeuvres and is zero-mean white Gaussian with covariance

(36) $$Q=\left[\begin{array}{cc} q_{x}B & 0\\ 0 & q_{y}B \end{array}\right],\;B=\left[\begin{array}{cc} T^{4}/4 & T^{3}/2\\ T^{3}/2 & T^{2} \end{array}\right]$$

where $q_{x}$ and $q_{y}$ are often determined from maximum target acceleration [28].

The AOA measurement equation is

(37) $$z_{k}=h\left(x_{k}\right)+w_{k}$$

where h(·) is the bearing angle of the target from the sensor location [see (2)], and $w_{k}∼N\left(0,\sigma^{2}\right)$ is the bearing measurement noise. As the measurement Equation (37) is nonlinear, the extended Kalman filter (EKF) is often used to estimate the target state vector, which is given by the recursion:

State Prediction:

(38) $$\begin{array}{cccc} \; & x_{k|k-1} & =Fx_{k-1|k-1} & \;\;\;\;\;\;\;\;\;\;\;\;\;\;\;\;\;\;\;\;\;\;\;\;\;\;\;\;\;\;\mathrm{mean}\;\mathrm{of}\;\mathrm{prior} \end{array}$$

(39) $$\begin{array}{ccc} P_{k|k-1} & =FP_{k-1|k-1}F^{T}+Q & \;\;\;\;\;\;\;\;\;\;\;\;\;\;\;\mathrm{covariance}\;\mathrm{of}\;\mathrm{prior} \end{array}$$

State Update:

(40) $$\begin{array}{ccc} \; & {\tilde{z}}_{k} & =z_{k}-h\left(x_{k|k-1}\right) \end{array}$$

(41) $$\begin{array}{ccc} \; & G_{k} & =P_{k|k-1}h_{k}^{T}{\left(h_{k}P_{k|k-1}h_{k}^{T}+\sigma^{2}\right)}^{-1} \end{array}$$

(42) $$\begin{array}{cccc} \; & x_{k|k} & =x_{k|k-1}+G_{k}{\tilde{z}}_{k} & \;\;\;\;\;\;\;\;\;\;\;\;\;\;\;\;\;\;\;\;\;\;\;\;\mathrm{state}\;\mathrm{estimate} \end{array}$$

(43) $$\begin{array}{cccc} \; & P_{k|k} & =\left(I-G_{k}h_{k}\right)P_{k|k-1} & \;\;\;\;\;\;\;\;\;\;\;\;\;\;\;\;\;\;\;\mathrm{covariance}\;\mathrm{of}\;\mathrm{state}\;\mathrm{estimate} \end{array}$$

where $x_{k|k-1}$ is the state prediction at time *k* given all measurements up to time k−1, and $x_{k|k}$ is the filtered state estimate at time *k*. The EKF replaces the nonlinear measurement Equation (37) with

(44) $$z_{k}=h_{k}x_{k}+w_{k}$$

where $h_{k}$ is the Jacobian of $h(x_{k})$ evaluated at $x_{k|k-1}$:

(45) $$h_{k}=\frac{1}{d_{k|k-1}}\left[\begin{array}{cccc} u_{1}\left(x_{k|k-1}\right) & 0 & u_{2}\left(x_{k|k-1}\right) & 0 \end{array}\right].$$

Here, $d_{k|k-1}$ is the target–sensor range estimate computed from $x_{k|k-1}$ and $s_{k}$, and $u\left(\cdot \right)={\left[u_{1}\left(\cdot \right),u_{2}\left(\cdot \right)\right]}^{T}$ is the unit vector defined in (4).

The moving AOA sensor is assumed to travel with a constant velocity, which means that the distance between successive waypoints is constant, i.e., $∥s_{k}-s_{k-1}∥=s$. Assuming a maximum turn-rate of $\pm \vartheta_{\max}$ in azimuth, the next waypoint is constrained to lie on an arc defined by

(46) $$s_{k}=s_{k-1}+s\;\upsilon \left(\vartheta_{k}\right),\;\left|\vartheta_{k}-\vartheta_{k-1}\right|<\vartheta_{\max}$$

where $\upsilon \left(\vartheta_{k}\right)={\left[\cos\vartheta_{k},\sin\vartheta_{k}\right]}^{T}$ is the sensor heading vector with heading angle $\vartheta_{k}$ at time instant *k*.

The recursive BFIM for the Kalman filter tracking problem is given by [24]

(47) $$\Phi_{k}={\left(F\Phi_{k-1}F^{T}+Q\right)}^{-1}+\frac{1}{\sigma^{2}}E_{x_{k}}\left\{{\tilde{h}}_{k}^{T}{\tilde{h}}_{k}\right\}$$

where ${\tilde{h}}_{k}$ is the Jacobian matrix in (45) calculated at target state $x_{k}$. Equation (47) has the same structure as (3) in that it is the sum of prior information ${\left(F\Phi_{k-1}F^{T}+Q\right)}^{-1}$ and contribution from measurements $\frac{1}{\sigma^{2}}E_{x_{k}}\left\{{\tilde{h}}_{k}^{T}{\tilde{h}}_{k}\right\}$. It is necessary to simplify (47) so that readily available Kalman filter estimates can be used rather than resorting to computationally expensive Monte Carlo simulations to calculate the expectation.

The contribution from measurements is approximated by

(48) $$\frac{1}{\sigma^{2}}E_{x_{k}}\left\{{\tilde{h}}_{k}^{T}{\tilde{h}}_{k}\right\}\approx \frac{1}{\sigma^{2}}h_{k}^{T}h_{k}$$

where $h_{k}$ is the Jacobian in (45). Thus, using $P_{k|k-1}^{-1}$ as the prior information and (48) as the contribution from measurements, we have

(49) $$\Phi_{k}\approx P_{k|k-1}^{-1}+\frac{1}{\sigma^{2}}h_{k}^{T}h_{k}$$

where both $P_{k|k-1}$ and $h_{k}$ are calculated by the EKF. In the following subsections, we show how to apply the D- and A-optimality criteria to the approximate recursive BFIM expression in (49) to derive trajectory optimization algorithms.

### 3.1. Sensor Trajectory Optimization Using D-Optimality

Referring to (21) and (49), the optimal waypoint for the sensor at time *k* using the D-optimality for the EKF takes the following form:

(50) $$\max_{s_{k}\in S_{k}}h_{k}P_{k|k-1}h_{k}^{T}$$

which can be rewritten as

(51) $$\max_{s_{k}\in S_{k}}\frac{1}{d_{k|k-1}^{2}}u^{T}\left(x_{k|k-1}\right)P_{\mathrm{loc},k|k-1}u\left(x_{k|k-1}\right)$$

where $S_{k}$ is the set of permissible waypoints compliant with the turn-rate

(52) $$S_{k}=\{s_{k}\;|\;s_{k}-s_{k-1}=s\upsilon \left(\vartheta_{k}\right),\;|\vartheta_{k}-\vartheta_{k-1}\left|<\vartheta_{\max}\right\}$$

and

(53) $$P_{\mathrm{loc},k|k-1}=\left[\begin{array}{cc} p_{\mathrm{loc},k|k-1}\left(1,1\right) & p_{\mathrm{loc},k|k-1}\left(1,2\right)\\ p_{\mathrm{loc},k|k-1}\left(2,1\right) & p_{\mathrm{loc},k|k-1}\left(2,2\right) \end{array}\right]$$

is the 2×2 covariance matrix for predicted target location, which is extracted from $P_{k|k-1}$ as shown below:

(54) $$P_{k|k-1}=\left[\begin{array}{cccc} p_{\mathrm{loc},k|k-1}\left(1,1\right) & * & p_{\mathrm{loc},k|k-1}\left(1,2\right) & *\\ {}* & * & * & *\\ p_{\mathrm{loc},k|k-1}\left(2,1\right) & * & p_{\mathrm{loc},k|k-1}\left(2,2\right) & *\\ {}* & * & * & * \end{array}\right].$$

Note that as $d_{k|k-1}$ also depends on $s_{k}$ (i.e., the fixed range constraint does not apply), (51) does not have a simple closed-form solution. This means that it must be solved by a numerical search over a finite number of permissible waypoints contained in the set $S_{k}$.

### 3.2. Sensor Trajectory Optimization Using A-Optimality

Using (28), the A-optimality criterion for sensor waypoints is given by

(55) $$\max_{s_{k}\in S_{k}}\frac{h_{k}^{T}P_{k|k-1}^{2}h_{k}}{\sigma^{2}+h_{k}^{T}P_{k|k-1}h_{k}}$$

which is obtained by substituting $h_{k}$ for $u^{T}\left(x_{0}\right)/d$ and $P_{k|k-1}$ for $P_{0}$ into (28).

Different from the D-optimality criterion in (51), (55) depends not only on the target range $d_{k|k-1}$ through the Jacobian $h_{k}$, but also the bearing noise variance $\sigma^{2}$. Again, it is not straightforward to solve (55) for the optimal $s_{k}$. A numerical search over the members of the set $S_{k}$ is necessary.

### 3.3. Projection Algorithm: A Closed-Form Solution

The D- and A-optimality solutions for determining optimal sensor waypoints described above can be computationally expensive, especially if the numerical search must be carried out over a large number of candidate waypoints in the set $S_{k}$. In this subsection, we present an alternative closed-form solution, called the projection algorithm, inspired by the ultimate objective of aligning the sensor with the minor axis of the prior covariance error ellipse.

**Figure 5:**
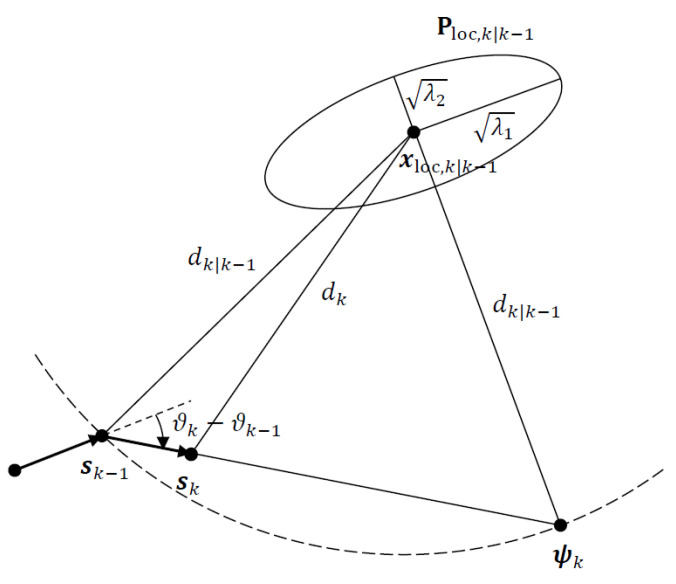
Closed-form projection algorithm to determine the next waypoint $s_{k}$. The heading angle $\vartheta_{k}$ is constrained by the turn-rate $|\vartheta_{k}-\vartheta_{k-1}|<\vartheta_{\max}$.

The idea behind the projection algorithm is illustrated in Figure 5. The next waypoint $s_{k}$ is chosen to guide the sensor towards the closest point $\psi_{k}$ that is aligned with the minor axis of the target location prior $P_{\mathrm{loc},k|k-1}$ and is at the same distance from the mean of the prior $x_{\mathrm{loc},k|k-1}={\left[x_{k|k-1},y_{k|k-1}\right]}^{T}$ as the estimated target–sensor range $d_{k|k-1}$. The next waypoint is found by projecting the waypoint vector $s_{k}-s_{k-1}$ to $\psi_{k}-s_{k-1}$ subject to the turn-rate constraint. If the projection causes the sensor heading angle to exceed the turn-rate, the next waypoint is chosen to have the maximum turn-rate. This projection also brings the sensor closer to the target with $d_{k}<d_{k|k-1}$. The reduction in $d_{k}$ is proportional to how far $\psi_{k}$ is from $s_{k-1}$. If $x_{\mathrm{loc},k|k-1}-s_{k-1}$ is aligned with the major axis of the error ellipse, which represents the worst geometry, the distance between $s_{k-1}$ and $\psi_{k}$ is maximized and $d_{k}$ will have the maximum reduction.

This behaviour makes sense because the optimality of a target–sensor geometry is improved at the maximum rate by moving the sensor directly towards the optimal sensor location for the given Gaussian prior with mean $x_{\mathrm{loc},k|k-1}$ and covariance $P_{\mathrm{loc},k|k-1}$.

A detailed description of the projection algorithm is provided in Algorithm 1.
**Algorithm 1** Projection algorithm.   **Input:** $s_{k-1}$, $x_{\mathrm{loc},k|k-1}$, $P_{\mathrm{loc},k|k-1}$, $\vartheta_{\max}$, $\vartheta_{k-1}$, *s*   
**Output:** 
$s_{k}$   Calculate $\nu_{2}$, eigenvector of $P_{\mathrm{loc},k|k-1}$ associated with its smallest eigenvalue   
Calculate $d_{k|k-1}=∥x_{\mathrm{loc},k|k-1}-s_{k-1}∥$   Calculate $\psi_{k}^{\pm }=x_{\mathrm{loc},k|k-1}\pm d_{k|k-1}\nu_{2}$   If $∥\psi_{k}^{+}-s_{k-1}∥<∥\psi_{k}^{-}-s_{k-1}∥$    $\psi_{k}=\psi_{k}^{+}$   Else    $\psi_{k}=\psi_{k}^{-}$   End   If $s_{k-1}=\psi_{k}$ (sensor is already aligned with prior covariance minor axis)    Return $s_{k}=s_{k-1}+s{\left[\cos\vartheta_{k-1},\sin\vartheta_{k-1}\right]}^{T}$ (no change in sensor heading)   Else    Calculate $\Delta s=s\frac{\psi_{k}-s_{k-1}}{∥\psi_{k}-s_{k-1}∥}$    Calculate $\Delta \vartheta =∠\Delta s-\vartheta_{k-1}$, −π≤Δϑ≤π (*∠* denotes bearing angle)    If $|\Delta \vartheta |<\vartheta_{\max}$     Return $s_{k}=s_{k-1}+\Delta s$    Else     Calculate $\vartheta_{k}=\vartheta_{k-1}+\mathrm{sign}\left(\Delta \vartheta \right)\vartheta_{\max}$     Return $s_{k}=s_{k-1}+s{\left[\cos\vartheta_{k},\sin\vartheta_{k}\right]}^{T}$    End   End


## 4. Simulation Examples

This section presents simulation examples to verify the optimization results and to demonstrate the performance of the sensor trajectory optimization algorithms developed in Section 2 and Section 3. In the first set of simulations, the focus is on optimal target–sensor geometries using the D- and A-optimality criteria for Bayesian target localization. The Gaussian prior has zero mean $x_{0}={\left[0,0\right]}^{T}$ and covariance

(56) $$P_{0}=\left[\begin{array}{cc} 27.6047 & -14.7721\\ -14.7721 & 22.3953 \end{array}\right]$$

with eigenvalues $\lambda_{1}=40$ and $\lambda_{2}=10$. The minor axis of the error ellipse make an angle of $50^{\circ }$ with the positive *x*-axis. The AOA sensor is allowed to be located on a circle of radius d=50 km centred at the mean of the Gaussian prior $x_{0}$, and the bearing angle noise standard deviation is $\sigma =5^{\circ }$.

**Figure 6:**
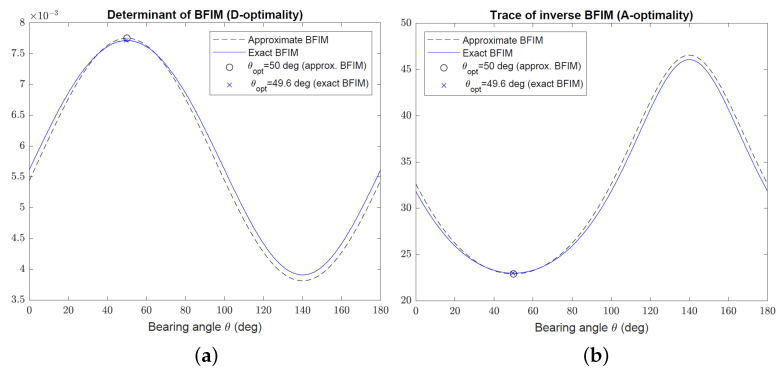
(**a**) Plot of determinant of Φ (D-optimality criterion) versus bearing angle using approximate and exact BFIM. (**b**) Plot of trace of $\Phi^{-1}$ (A-optimality criterion) versus bearing angle using approximate and exact BFIM.

Figure 6 shows the D- and A-optimality measures, |Φ| and $\mathrm{tr}\Phi^{-1}$, respectively, versus the bearing angle θ in the range 0≤θ<π for the approximate and exact BFIM given by (9) and (3), respectively. The exact BFIM was calculated using 50,000 Monte Carlo runs for each bearing angle. As evident from Figure 6, the simulated optimal bearing angles are not significantly different for the approximate and exact BFIM. This is also backed up by the close proximity of the two curves in Figure 6. The optimal bearing angle obtained from the approximate BFIM aligns with the minor axis of the error ellipse at $\theta_{\mathrm{opt}}=50^{\circ }$ for both D- and A-optimality criteria, which is in agreement with the analytical results derived in Section 2.

**Figure 7:**
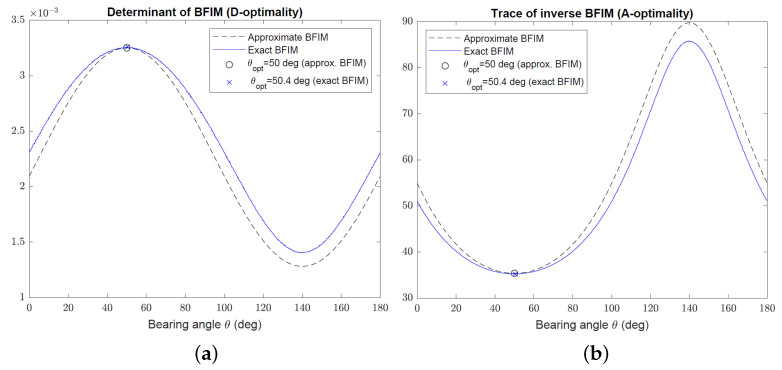
(**a**) Plot of determinant of Φ (D-optimality criterion) versus bearing angle for large $P_{0}$ using approximate and exact BFIM. (**b**) Plot of trace of $\Phi^{-1}$ (A-optimality criterion) versus bearing angle for large $P_{0}$ using approximate and exact BFIM.

To confirm that (9) is a valid approximation only for sufficiently small $P_{0}$ compared with the target range, we repeated the previous simulations for $P_{0}$ increased by a factor of two. Figure 7 shows the resulting D- and A-optimality measures for the approximate and exact BFIM. While the approximate and exact BFIM still yield almost the same optimal bearing angles, the curves corresponding to them exhibit significant discrepancy, in particular at bearing angles away from $\theta_{\mathrm{opt}}$.

**Figure 8:**
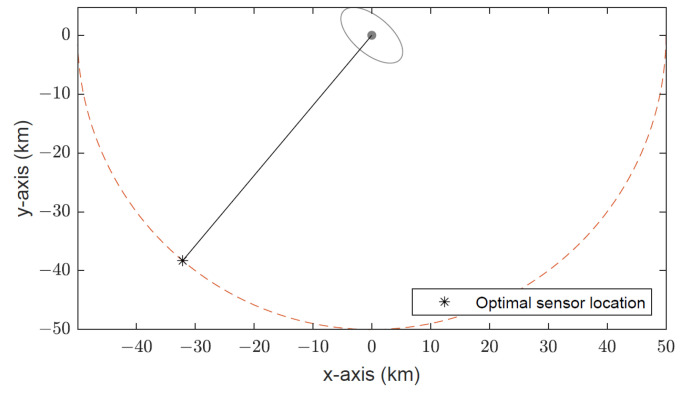
Optimal target–sensor geometry, where the target–sensor range vector is perfectly aligned with the minor axis of the error ellipse of the Gaussian prior covariance.

The optimal target–sensor geometry for the simulated scenario is depicted in Figure 8. As expected, at the optimal bearing angle, the target–sensor range vector is perfectly aligned with the minor axis of the error ellipse of the Gaussian prior. Note that there are, in fact, two optimal sensor locations. The other one has the bearing angle $\theta_{\mathrm{opt}}-180=-130$ degrees.

In the next set of simulations, we consider sensor trajectory optimization in a bearings-only manoeuvring target tracking problem. The algorithms in (51), (55) and Algorithm 1 are simulated for a single realization of target manoeuvres. The process noise parameter for the target is $q_{x}=q_{y}=10^{-4}$ m${}^{2}$/s${}^{4}$. The bearing angle noise is assumed to be $\sigma =4^{\circ }$. The initial target dynamics are

(57) $$x_{0|-1}={\left[0,0,0,0\right]}^{T}$$

and

(58) $$P_{0|-1}=\left[\begin{array}{cccc} 19.7500 & 0 & 9.0933 & 0\\ 0 & 0 & 0 & 0\\ 9.0933 & 0 & 9.2500 & 0\\ 0 & 0 & 0 & 0 \end{array}\right].$$

The time interval between successive EKF recursions is set equal to T=10 s. The sensor is initially located at $s_{0}=\left[-26.8116,-22.4976\right]$ km and moves with a constant speed of 25 m/s (90 km/h). The maximum turn-rate for the sensor is $30^{\circ }$ per 10 s. The separation between successive sensor waypoints is s=0.25 km. For the sensor trajectory optimization methods based on the D- and A-optimality criteria, 10 uniformly spaced points are used for numerical search over sensor waypoints in the set $S_{k}$.

**Figure 9:**
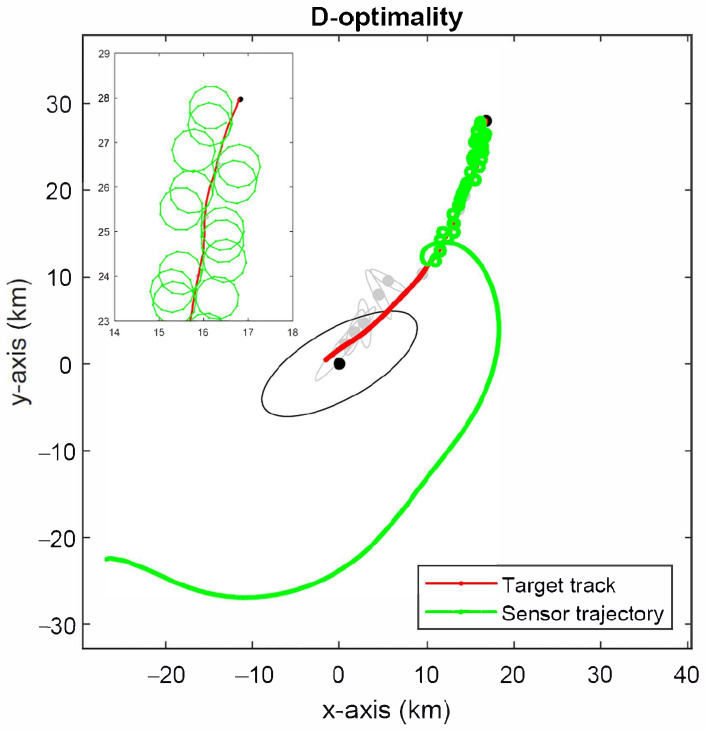
Optimal sensor trajectory for D-optimality criterion.

The simulated sensor trajectory for the D-optimality criterion in (51) is depicted in Figure 9. The 2-σ error ellipses for predicted target location estimates are plotted every 50 time instants. The initial error ellipse is drawn in black and all others are in grey. The optimal sensor trajectory achieves a rapid reduction in the size of error ellipses. Following the initial approach, the sensor chases the target by circling around it.

**Figure 10:**
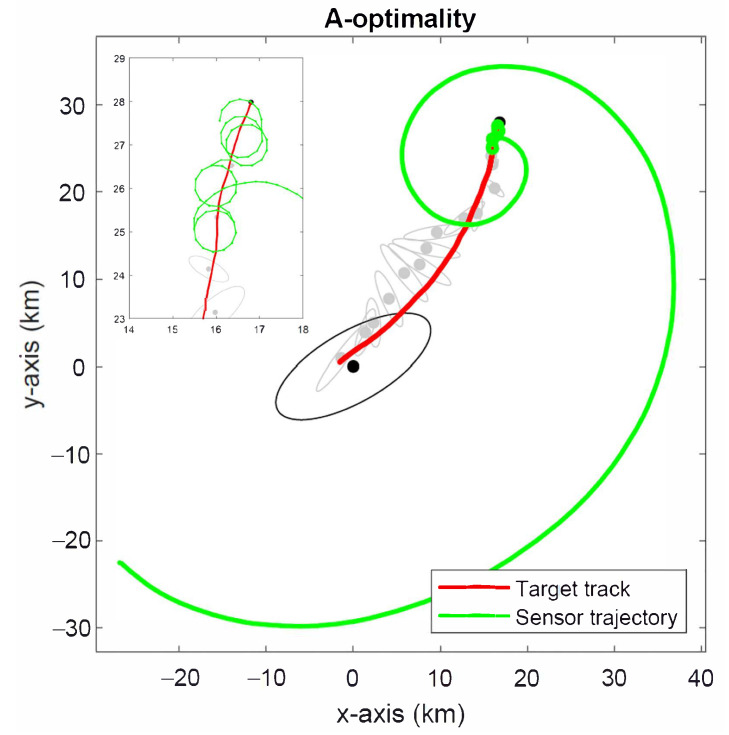
Optimal sensor trajectory for A-optimality criterion.

Figure 10 shows the simulated sensor trajectory for the A-optimality criterion in (55). The optimal sensor trajectory has a markedly different behaviour to that observed for the D-optimality criterion (see Figure 9) in that it seems to favour circling the target more than getting close to it initially. As a consequence, it takes longer to achieve a significant reduction in error ellipses than the A-optimality criterion.

**Figure 11:**
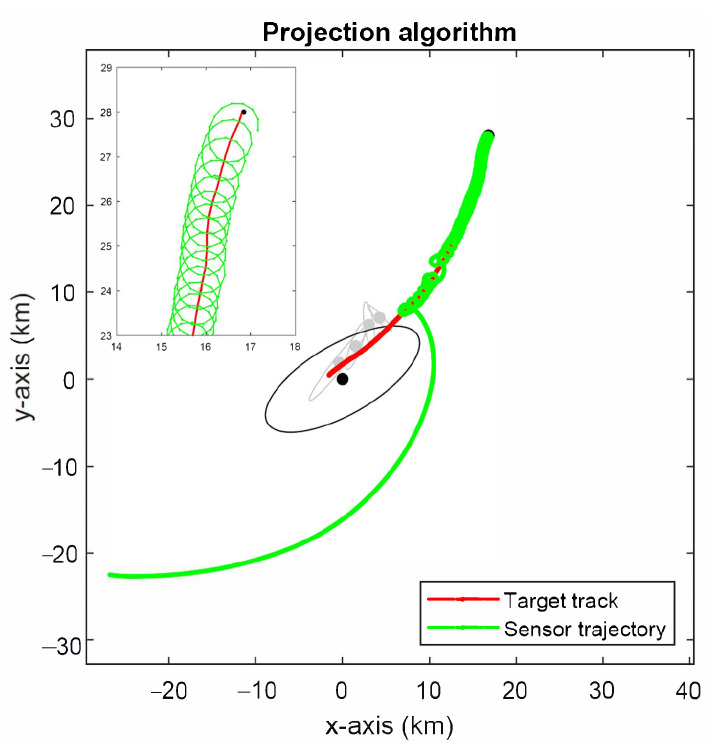
Optimal sensor trajectory using the projection algorithm.

**Figure 12:**
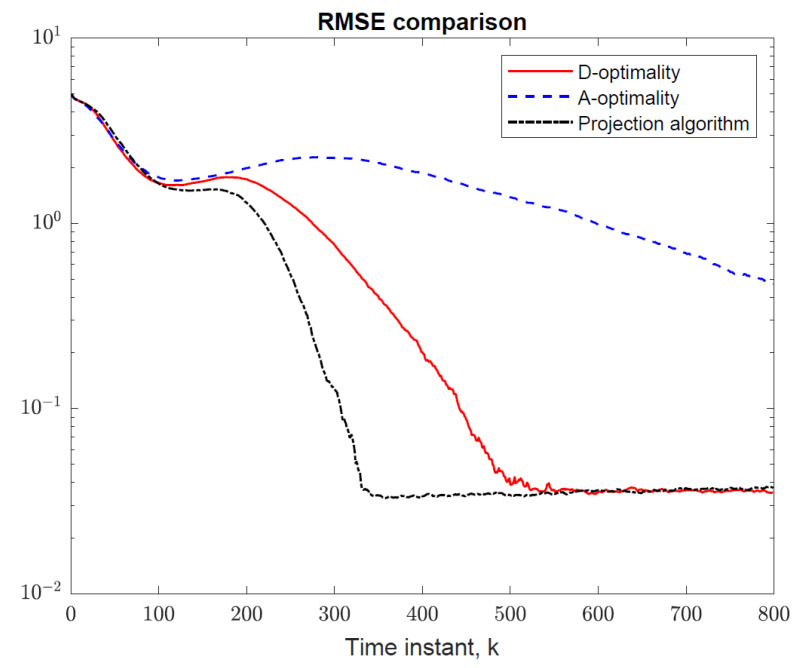
RMSE comparison of sensor trajectory optimization methods. The projection algorithm minimizes target tracking error fastest, followed by the D-optimality method.

The simulation results for the projection algorithm in Algorithm 1 are shown in Figure 11. The sensor follows a more direct route towards the target than both the D- and A-optimality methods, followed by circling manoeuvres. This is expected to produce faster convergence to the minimum estimation error than the D- and A-optimality methods at the expense of somewhat larger estimation error initially. This observation is confirmed by the root-mean-square error (RMSE) of target location estimates shown in Figure 12, which were computed from 5000 Monte Carlo simulation runs.

## 5. Conclusions

Optimal sensor placement for the Bayesian AOA target localization problem was considered. The concept of updating prior information with data from measurements was extended to the Kalman filter tracking problem. Using an approximation of the BFIM, the optimal sensor placement for a given Gaussian prior was shown to be aligned with the minor axis of the prior error ellipse for both D- and A-optimality criteria. By way of simulations, this result was shown to match the optimal sensor placement result for the exact BFIM, which was numerically calculated using Monte Carlo simulations. The D- and A-optimality criteria were adopted for optimal sensor guidance in target tracking applications. Simple methods requiring a numerical search over sensor heading were developed and demonstrated in simulations. A new method, called the projection algorithm, was also developed predicated on the optimal sensor placement result for the D- and A-optimality criteria. The efficacy of the projection algorithm in achieving fast minimization of tracking error was confirmed by numerical simulations. Even though the D- and A-optimality criteria share the same optimal sensor placement result for a stationary target, they generate quite different sensor trajectories in a target tracking setting where optimal target–sensor geometries cannot be realized instantaneously because of constrained sensor dynamics. The A-optimality method was observed to favour circular motion around the target initially. This results in delays in minimizing the tracking error to a desirable level.

## Data Availability

Not applicable.
